# Poly[bis­[μ-1,4-bis­(1*H*-imidazol-5-yl)benzene-κ^2^ *N* ^3^:*N* ^3′^]diformatomanganese(II)]

**DOI:** 10.1107/S1600536810044053

**Published:** 2010-11-06

**Authors:** Shui-Sheng Chen, Sen-Lin Yang, Shu-Ping Zhang, Zi-Qiang Lu

**Affiliations:** a Department of Chemistry, Fuyang Normal College, Fuyang, Anhui 236041, People’s Republic of China; b Key Laboratory of Functional Organometallic Materials, Department of Chemistry and Materials Science, Hengyang Normal University, Luoyang, Henan 471022, People’s Republic of China

## Abstract

In the title compound, [Mn(CHO_2_)_2_(C_12_H_10_N_4_)_2_]_*n*_, the Mn^II^ atom and the benzene ring of the ligand lie on an inversion centers. The Mn^II^ atom has an octa­hedral coordination environment composed of four N atoms from two different symmetry-related *N*-heterocyclic ligands forming the basal plane, and two O atoms from symmetry-related formate anions occupying the apical positions. The title compound forms a two-dimensional (4,4) net parallel to (100) with all the Mn^II^ atoms lying on a plane. The crystal structure is consolidated by inter­molecular N—H⋯O hydrogen bonds..

## Related literature

For related literature on transition metal complex assembly, see: Kitagawa & Kondo (1998 ▶). For related literature on novel coordination networks belonging to entangled systems, see: Batten & Robson (1998 ▶); Hoskins *et al.* (1997*a* ▶,*b* ▶). For a related Mn^II^ complex, see: Zhao *et al.* (2009 ▶); Zhu *et al.* (2010 ▶). For three-dimensional structures, see: Tian *et al.* (2007 ▶). 
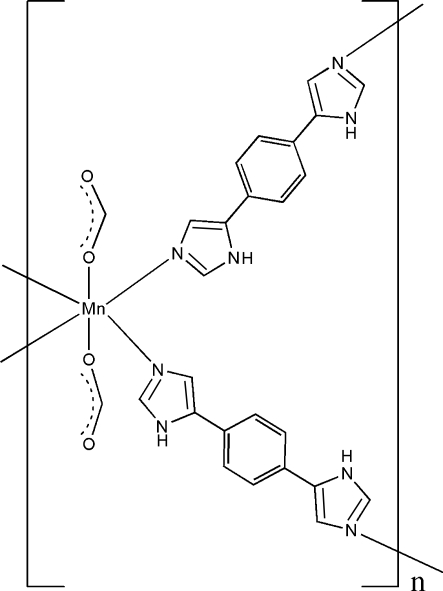


## Experimental

### 

#### Crystal data

[Mn(CHO_2_)_2_(C_12_H_10_N_4_)_2_]
*M* *_r_* = 565.46
Monoclinic, 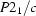

*a* = 7.3240 (8) Å
*b* = 12.1313 (13) Å
*c* = 14.1802 (15) Å
β = 100.704 (2)°
*V* = 1238.0 (2) Å^3^
*Z* = 2
Mo *K*α radiation
μ = 0.59 mm^−1^
*T* = 293 K
0.21 × 0.16 × 0.12 mm

#### Data collection

Bruker SMART APEXII CCD diffractometer
Absorption correction: multi-scan (*SADABS*; Sheldrick, 1996 ▶) *T*_min_ = 0.887, *T*_max_ = 0.933
6495 measured reflections
2420 independent reflections
2196 reflections with *I* > 2σ(*I*)
*R*_int_ = 0.043

#### Refinement

*R*[*F* ^2^ > 2σ(*F* ^2^)] = 0.036
*wR*(*F* ^2^) = 0.101
*S* = 1.06
2420 reflections
178 parameters
H-atom parameters constrained
Δρ_max_ = 0.30 e Å^−3^
Δρ_min_ = −0.25 e Å^−3^

### 

Data collection: *APEX2* (Bruker, 2003 ▶); cell refinement: *SAINT* (Bruker, 2003 ▶); data reduction: *SAINT*; program(s) used to solve structure: *SHELXS97* (Sheldrick, 2008 ▶); program(s) used to refine structure: *SHELXL97* (Sheldrick, 2008 ▶); molecular graphics: *SHELXTL* (Sheldrick, 2008 ▶); software used to prepare material for publication: *SHELXTL*.

## Supplementary Material

Crystal structure: contains datablocks global, I. DOI: 10.1107/S1600536810044053/bx2313sup1.cif

Structure factors: contains datablocks I. DOI: 10.1107/S1600536810044053/bx2313Isup2.hkl

Additional supplementary materials:  crystallographic information; 3D view; checkCIF report

## Figures and Tables

**Table 1:** Hydrogen-bond geometry (Å, °)

*D*—H⋯*A*	*D*—H	H⋯*A*	*D*⋯*A*	*D*—H⋯*A*
N2—H2⋯O2^i^	0.86	2.00	2.840 (2)	165
N4—H4⋯O2^ii^	0.86	1.95	2.736 (2)	151

Symmetry codes: (i) 
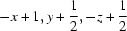
; (ii) 
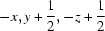
.

**Fig. 1.**
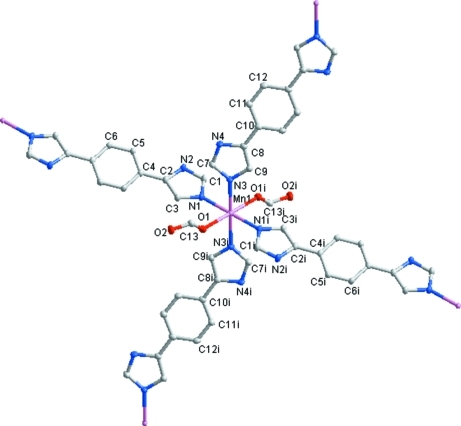
The ORTEP drawing of the title compound (I). Displacement ellipsoids are drawn at 30% probability level. Symmetry codes: (i) 1-x, 1-y, 1-z.

**Fig. 2.**
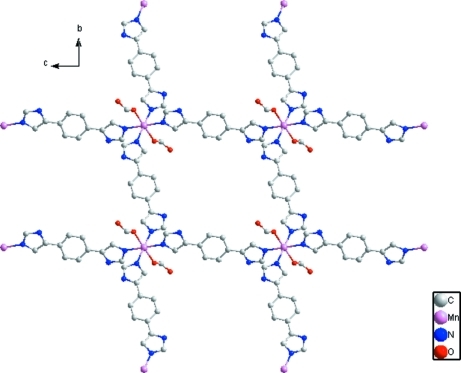
Two-dimensional (4,4) rectangular grid [Mn(L)2]n coordination polymer layer motif along the bc plane of the compound (I).

**Fig. 3.**
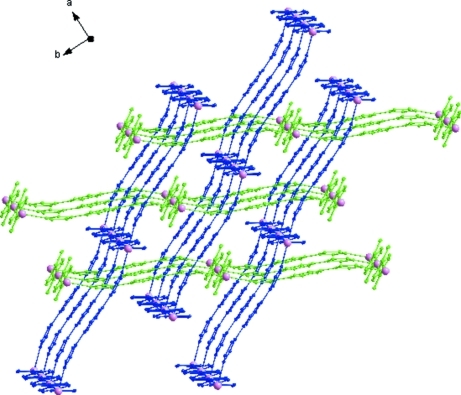
The three-dimensional structure built from two-dimension mutual inclined interpenetration of the compound (I).

## supplementary crystallographic information

### Comment

Recently, a great deal of interest in transition metal complex assembly has been devoted to the development of rational synthetic routes to novel crystal frameworks, due to their potential applications in many areas (Kitagawa *et al.*, 1998). So far large quantities of novel coordination networks belonging to entangled systems have been reported in the literature (Batten *et al.*, 1998). It has been demostrated that the flexible bridging ligand can easily construct entangled systems. For example, 1,4-bix(imidazol-1-yl-methyl) benzene (bix) formed an infinite polyrotaxane network of [Ag_2_(bix)_3_(NO_3_)_2_] by reactions with silver nitrate (Hoskins *et al.*, 1997*a*) and gave a two-dimensional interpenetrated network of [Zn(bix)_2_(NO_3_)_2_.4.5H_2_O] by reactions with zinc nitrate which has both polyrotaxane and polycatenane characters (Hoskins *et al.*, 1997*b*). As an extension of above work, we report a new entangled metal complex [Mn(C_24_H_20_N_8_)_2_(HCOO)_2_]_n_ (I) based on rigid 1,4-di(1*H*-imidazol-4-yl)benzene ligand (*L*) and metal Mn^II^ salts. In the title compound, the Mn ^II^ atom and the ring benzene of the ligand  are lies on inversion center. The Mn^II^ has an octahedral coordination environment surrounded by four nitrogen atoms from two different N-heterocyclic ligands symmetry-related forming the basal plane and two oxygen donors from one formate anion symmetry-related occupying the apical positions (Fig. 1). The Mn—N distances are comparable to those found in other crystallographically characterized Mn^II^ complex (Zhao *et al.*, 2009) and Mn—O distance is coincident with another Mn^II^ complex (Zhu *et al.*, 2010). The title compound form two-dimensional (4,4) net and its building unit is [Mn(*L*)]. The Mn^II^ are connected to a 1D linear chain along b-axis. The ligand, L, link Mn^II ^ions in adjacent chains by the same mode as described above, which makes the Mn^II ^ions links another 1D chain along c-axis. Therefore, the title compound is further connect to a 2D infinite strucure in bc plane, Fig. 2. The void spaces within the [Mn(*L*)_2_]_n_ coordination polymer layers permit mutual inclined two parallel sets of layers to angle into three dimensional framework (Tian *et al.*, 2007) (Fig. 3). The crystal structure of the title compound is stabilized by two intermolecular N—H···O interactions with average H···O distances 2.00 Å and N—H···O angles in the range 151-165°.

### Experimental

All reagents and solvents were used as obtained commercially without further purification. A mixture containing MnCl_2_.4H_2_O (19.8 mg, 0.05 mmol), *L* (27.6 mg, 0.1 mmol), DMF (N:*N*'- dimethylformamide, 1 mL), 10 ml H_2_O was sealed in a 16 ml Teflon-lined stainless steel container and heated at 393 K for 72 h. After cooling to room temperature within 12 h, block brown crystals of (I) suitable for X-ray diffraction analysis were obtained in 69% Yield.

### Refinement

H atoms bonded to C atoms were placed geometrically and treated as riding, with C—H distances 0.93 Å and *U*_iso_(H) = 1.2*U*_eq_(C). The amide H atoms were located from difference maps and refined with the N—H distances restrained to 0.86 Å and *U*_iso_(H) = 1.2*U*_eq_(N).

### Crystal data

**Table:**

[Mn(CHO_2_)_2_(C_24_H_20_N_8_)_2_]	*F*(000) = 582
*M**_r_* = 565.46	*D*_x_ = 1.517 Mg m^−^^3^
Monoclinic, *P*2_1_/*c*	Mo *K*α radiation, λ = 0.71073 Å
Hall symbol: -P 2ybc	Cell parameters from 3510 reflections
*a* = 7.3240 (8) Å	θ = 2.2–28.2°
*b* = 12.1313 (13) Å	µ = 0.59 mm^−^^1^
*c* = 14.1802 (15) Å	*T* = 293 K
β = 100.704 (2)°	Block, brown
*V* = 1238.0 (2) Å^3^	0.21 × 0.16 × 0.12 mm
*Z* = 2	

### Data collection

**Table:**

Bruker SMART APEXII CCD diffractometer	2420 independent reflections
Radiation source: fine-focus sealed tube	2196 reflections with *I* > 2σ(*I*)
graphite	*R*_int_ = 0.043
φ and ω scans	θ_max_ = 26.0°, θ_min_ = 2.2°
Absorption correction: multi-scan (*SADABS*; Sheldrick, 1996)	*h* = −9→4
*T*_min_ = 0.887, *T*_max_ = 0.933	*k* = −14→14
6495 measured reflections	*l* = −17→17

### Refinement

**Table:**

Refinement on *F*^2^	Primary atom site location: structure-invariant direct methods
Least-squares matrix: full	Secondary atom site location: difference Fourier map
*R*[*F*^2^ > 2σ(*F*^2^)] = 0.036	Hydrogen site location: inferred from neighbouring sites
*wR*(*F*^2^) = 0.101	H-atom parameters constrained
*S* = 1.06	*w* = 1/[σ^2^(*F*_o_^2^) + (0.0581*P*)^2^ + 0.230*P*] where *P* = (*F*_o_^2^ + 2*F*_c_^2^)/3
2420 reflections	(Δ/σ)_max_ < 0.001
178 parameters	Δρ_max_ = 0.30 e Å^−^^3^
0 restraints	Δρ_min_ = −0.25 e Å^−^^3^

### Special details

**Table:**

Geometry. All e.s.d.'s (except the e.s.d. in the dihedral angle between two l.s. planes) are estimated using the full covariance matrix. The cell e.s.d.'s are taken into account individually in the estimation of e.s.d.'s in distances, angles and torsion angles; correlations between e.s.d.'s in cell parameters are only used when they are defined by crystal symmetry. An approximate (isotropic) treatment of cell e.s.d.'s is used for estimating e.s.d.'s involving l.s. planes.
Refinement. Refinement of *F*^2^ against ALL reflections. The weighted *R*-factor *wR* and goodness of fit *S* are based on *F*^2^, conventional *R*-factors *R* are based on *F*, with *F* set to zero for negative *F*^2^. The threshold expression of *F*^2^ > σ(*F*^2^) is used only for calculating *R*-factors(gt) *etc*. and is not relevant to the choice of reflections for refinement. *R*-factors based on *F*^2^ are statistically about twice as large as those based on *F*, and *R*- factors based on ALL data will be even larger.

### Fractional atomic coordinates and isotropic or equivalent isotropic displacement parameters (Å^2^)

**Table:**

	*x*	*y*	*z*	*U*_iso_*/*U*_eq_	
C1	0.7024 (3)	0.61227 (17)	0.34180 (14)	0.0363 (5)	
H1	0.7649	0.6613	0.3869	0.044*	
C2	0.5906 (3)	0.53538 (16)	0.20304 (13)	0.0292 (4)	
C3	0.5424 (3)	0.47633 (17)	0.27654 (14)	0.0319 (5)	
H3	0.4721	0.4120	0.2690	0.038*	
C4	0.5462 (3)	0.51810 (15)	0.09923 (14)	0.0265 (4)	
C5	0.5767 (3)	0.59952 (16)	0.03487 (13)	0.0289 (4)	
H5	0.6284	0.6666	0.0576	0.035*	
C6	0.5306 (3)	0.58109 (16)	−0.06262 (13)	0.0298 (4)	
H6	0.5514	0.6364	−0.1048	0.036*	
C7	0.2276 (3)	0.66265 (16)	0.35944 (15)	0.0386 (5)	
H7	0.2459	0.6234	0.3056	0.046*	
C8	0.1345 (3)	0.79075 (15)	0.44854 (14)	0.0292 (4)	
C9	0.2315 (3)	0.71098 (15)	0.50414 (14)	0.0323 (4)	
H9	0.2541	0.7105	0.5709	0.039*	
C10	0.0609 (3)	0.89682 (15)	0.47356 (14)	0.0290 (4)	
C11	−0.0078 (3)	0.97491 (17)	0.40435 (16)	0.0351 (5)	
H11	−0.0133	0.9588	0.3398	0.042*	
C12	−0.0679 (3)	1.07637 (16)	0.43069 (15)	0.0355 (5)	
H12	−0.1139	1.1276	0.3834	0.043*	
C13	0.1608 (3)	0.36826 (16)	0.37714 (14)	0.0339 (5)	
H13	0.0769	0.4215	0.3893	0.041*	
Mn1	0.5000	0.5000	0.5000	0.02250 (15)	
N1	0.6124 (3)	0.52515 (14)	0.36355 (12)	0.0330 (4)	
N2	0.6934 (3)	0.62227 (14)	0.24675 (11)	0.0351 (4)	
H2	0.7431	0.6738	0.2183	0.042*	
N3	0.2911 (2)	0.63150 (13)	0.44833 (12)	0.0317 (4)	
N4	0.1335 (2)	0.75759 (14)	0.35583 (12)	0.0350 (4)	
H4	0.0817	0.7917	0.3047	0.042*	
O1	0.3183 (2)	0.37154 (11)	0.42634 (10)	0.0378 (4)	
O2	0.1041 (2)	0.30061 (13)	0.31300 (11)	0.0461 (4)	

### Atomic displacement parameters (Å^2^)

**Table:**

	*U*^11^	*U*^22^	*U*^33^	*U*^12^	*U*^13^	*U*^23^
C1	0.0449 (12)	0.0407 (11)	0.0220 (10)	−0.0042 (10)	0.0024 (9)	−0.0056 (8)
C2	0.0326 (10)	0.0325 (10)	0.0223 (10)	0.0011 (8)	0.0050 (8)	−0.0028 (8)
C3	0.0420 (12)	0.0318 (10)	0.0222 (10)	−0.0031 (9)	0.0068 (9)	−0.0030 (8)
C4	0.0282 (10)	0.0307 (9)	0.0206 (9)	0.0025 (8)	0.0048 (8)	−0.0009 (7)
C5	0.0344 (11)	0.0270 (9)	0.0245 (10)	−0.0020 (8)	0.0035 (8)	−0.0031 (7)
C6	0.0363 (11)	0.0309 (10)	0.0225 (9)	−0.0007 (8)	0.0061 (8)	0.0037 (7)
C7	0.0481 (13)	0.0319 (11)	0.0323 (11)	0.0115 (10)	−0.0014 (9)	−0.0050 (9)
C8	0.0278 (10)	0.0256 (9)	0.0326 (10)	0.0020 (8)	0.0016 (8)	−0.0003 (8)
C9	0.0405 (11)	0.0295 (10)	0.0261 (10)	0.0054 (9)	0.0037 (8)	0.0000 (8)
C10	0.0247 (9)	0.0247 (9)	0.0367 (11)	0.0029 (7)	0.0031 (8)	0.0002 (8)
C11	0.0402 (12)	0.0338 (10)	0.0305 (10)	0.0067 (9)	0.0044 (9)	−0.0008 (8)
C12	0.0394 (12)	0.0306 (10)	0.0346 (11)	0.0096 (9)	0.0024 (9)	0.0069 (8)
C13	0.0388 (12)	0.0284 (10)	0.0331 (11)	0.0017 (9)	0.0029 (9)	−0.0045 (8)
Mn1	0.0303 (3)	0.0201 (2)	0.0153 (2)	0.00383 (15)	−0.00031 (16)	−0.00102 (13)
N1	0.0423 (10)	0.0362 (9)	0.0204 (8)	−0.0006 (8)	0.0059 (7)	−0.0017 (7)
N2	0.0465 (10)	0.0377 (9)	0.0210 (8)	−0.0095 (8)	0.0056 (7)	−0.0013 (7)
N3	0.0395 (10)	0.0266 (8)	0.0267 (8)	0.0088 (7)	0.0000 (7)	−0.0006 (6)
N4	0.0411 (10)	0.0305 (9)	0.0285 (9)	0.0102 (7)	−0.0062 (7)	0.0025 (7)
O1	0.0427 (9)	0.0303 (8)	0.0348 (8)	−0.0038 (6)	−0.0075 (7)	−0.0042 (6)
O2	0.0523 (10)	0.0427 (9)	0.0351 (9)	−0.0002 (7)	−0.0131 (7)	−0.0127 (7)

### Geometric parameters (Å, °)

**Table:**

C1—N1	1.312 (3)	C9—N3	1.369 (2)
C1—N2	1.343 (3)	C9—H9	0.9300
C1—H1	0.9300	C10—C12^ii^	1.388 (3)
C2—C3	1.364 (3)	C10—C11	1.389 (3)
C2—N2	1.375 (3)	C11—C12	1.382 (3)
C2—C4	1.462 (3)	C11—H11	0.9300
C3—N1	1.379 (3)	C12—C10^ii^	1.388 (3)
C3—H3	0.9300	C12—H12	0.9300
C4—C6^i^	1.388 (3)	C13—O1	1.233 (2)
C4—C5	1.391 (3)	C13—O2	1.238 (2)
C5—C6	1.379 (3)	C13—H13	0.9300
C5—H5	0.9300	Mn1—O1	2.1848 (13)
C6—C4^i^	1.388 (3)	Mn1—O1^iii^	2.1848 (13)
C6—H6	0.9300	Mn1—N3^iii^	2.2376 (16)
C7—N3	1.315 (3)	Mn1—N3	2.2376 (16)
C7—N4	1.338 (2)	Mn1—N1	2.2597 (17)
C7—H7	0.9300	Mn1—N1^iii^	2.2597 (17)
C8—C9	1.362 (3)	N2—H2	0.8600
C8—N4	1.373 (3)	N4—H4	0.8600
C8—C10	1.464 (3)		
			
N1—C1—N2	112.07 (18)	C11—C12—H12	119.4
N1—C1—H1	124.0	C10^ii^—C12—H12	119.4
N2—C1—H1	124.0	O1—C13—O2	126.0 (2)
C3—C2—N2	104.84 (17)	O1—C13—H13	117.0
C3—C2—C4	130.79 (19)	O2—C13—H13	117.0
N2—C2—C4	124.36 (17)	O1—Mn1—O1^iii^	180.0
C2—C3—N1	110.64 (18)	O1—Mn1—N3^iii^	88.09 (6)
C2—C3—H3	124.7	O1^iii^—Mn1—N3^iii^	91.91 (6)
N1—C3—H3	124.7	O1—Mn1—N3	91.91 (6)
C6^i^—C4—C5	118.28 (17)	O1^iii^—Mn1—N3	88.09 (6)
C6^i^—C4—C2	119.98 (17)	N3^iii^—Mn1—N3	180.00 (8)
C5—C4—C2	121.74 (17)	O1—Mn1—N1	88.47 (6)
C6—C5—C4	120.23 (18)	O1^iii^—Mn1—N1	91.53 (6)
C6—C5—H5	119.9	N3^iii^—Mn1—N1	92.34 (6)
C4—C5—H5	119.9	N3—Mn1—N1	87.66 (6)
C5—C6—C4^i^	121.50 (17)	O1—Mn1—N1^iii^	91.53 (6)
C5—C6—H6	119.3	O1^iii^—Mn1—N1^iii^	88.47 (6)
C4^i^—C6—H6	119.3	N3^iii^—Mn1—N1^iii^	87.66 (6)
N3—C7—N4	111.81 (18)	N3—Mn1—N1^iii^	92.34 (6)
N3—C7—H7	124.1	N1—Mn1—N1^iii^	180.0
N4—C7—H7	124.1	C1—N1—C3	104.77 (17)
C9—C8—N4	104.81 (16)	C1—N1—Mn1	126.06 (13)
C9—C8—C10	131.31 (18)	C3—N1—Mn1	125.06 (14)
N4—C8—C10	123.55 (17)	C1—N2—C2	107.68 (16)
C8—C9—N3	110.71 (17)	C1—N2—H2	126.2
C8—C9—H9	124.6	C2—N2—H2	126.2
N3—C9—H9	124.6	C7—N3—C9	104.94 (16)
C12^ii^—C10—C11	118.30 (18)	C7—N3—Mn1	128.10 (14)
C12^ii^—C10—C8	119.65 (17)	C9—N3—Mn1	125.79 (13)
C11—C10—C8	121.96 (19)	C7—N4—C8	107.72 (17)
C12—C11—C10	120.5 (2)	C7—N4—H4	126.1
C12—C11—H11	119.7	C8—N4—H4	126.1
C10—C11—H11	119.7	C13—O1—Mn1	135.75 (13)
C11—C12—C10^ii^	121.16 (18)		
			
N2—C2—C3—N1	0.3 (2)	O1^iii^—Mn1—N1—C3	−172.33 (16)
C4—C2—C3—N1	−178.5 (2)	N3^iii^—Mn1—N1—C3	95.69 (16)
C3—C2—C4—C6^i^	−12.5 (3)	N3—Mn1—N1—C3	−84.31 (16)
N2—C2—C4—C6^i^	168.97 (19)	N1—C1—N2—C2	−0.1 (2)
C3—C2—C4—C5	166.7 (2)	C3—C2—N2—C1	−0.1 (2)
N2—C2—C4—C5	−11.9 (3)	C4—C2—N2—C1	178.79 (19)
C6^i^—C4—C5—C6	0.2 (3)	N4—C7—N3—C9	0.7 (2)
C2—C4—C5—C6	−178.98 (18)	N4—C7—N3—Mn1	−167.33 (14)
C4—C5—C6—C4^i^	−0.2 (3)	C8—C9—N3—C7	−0.9 (2)
N4—C8—C9—N3	0.8 (2)	C8—C9—N3—Mn1	167.49 (13)
C10—C8—C9—N3	−172.7 (2)	O1—Mn1—N3—C7	−63.64 (19)
C9—C8—C10—C12^ii^	−7.5 (3)	O1^iii^—Mn1—N3—C7	116.36 (19)
N4—C8—C10—C12^ii^	−179.89 (19)	N1—Mn1—N3—C7	24.74 (18)
C9—C8—C10—C11	169.0 (2)	N1^iii^—Mn1—N3—C7	−155.26 (18)
N4—C8—C10—C11	−3.4 (3)	O1—Mn1—N3—C9	130.62 (16)
C12^ii^—C10—C11—C12	−0.2 (3)	O1^iii^—Mn1—N3—C9	−49.38 (16)
C8—C10—C11—C12	−176.81 (19)	N1—Mn1—N3—C9	−140.99 (17)
C10—C11—C12—C10^ii^	0.3 (4)	N1^iii^—Mn1—N3—C9	39.01 (17)
N2—C1—N1—C3	0.3 (2)	N3—C7—N4—C8	−0.3 (2)
N2—C1—N1—Mn1	−157.68 (15)	C9—C8—N4—C7	−0.3 (2)
C2—C3—N1—C1	−0.3 (2)	C10—C8—N4—C7	173.77 (19)
C2—C3—N1—Mn1	157.90 (14)	O2—C13—O1—Mn1	151.13 (17)
O1—Mn1—N1—C1	161.35 (18)	N3^iii^—Mn1—O1—C13	178.4 (2)
O1^iii^—Mn1—N1—C1	−18.65 (18)	N3—Mn1—O1—C13	−1.6 (2)
N3^iii^—Mn1—N1—C1	−110.62 (18)	N1—Mn1—O1—C13	−89.2 (2)
N3—Mn1—N1—C1	69.38 (18)	N1^iii^—Mn1—O1—C13	90.8 (2)
O1—Mn1—N1—C3	7.67 (16)		

Symmetry codes: (i) −*x*+1, −*y*+1, −*z*; (ii) −*x*, −*y*+2, −*z*+1; (iii) −*x*+1, −*y*+1, −*z*+1.

### Hydrogen-bond geometry (Å, °)

**Table:**

*D*—H···*A*	*D*—H	H···*A*	*D*···*A*	*D*—H···*A*
N2—H2···O2^iv^	0.86	2.00	2.840 (2)	165
N4—H4···O2^v^	0.86	1.95	2.736 (2)	151

Symmetry codes: (iv) −*x*+1, *y*+1/2, −*z*+1/2; (v) −*x*, *y*+1/2, −*z*+1/2.
